# Supplementary material for: Comparative Efficacy of ALK Inhibitors for Treatment-Naïve ALK-Positive Advanced Non-Small Cell Lung Cancer with Central Nervous System Metastasis: A Network Meta-Analysis
Source: Int J Mol Sci. 2023 Jan 23;24(3):2242. doi: 10.3390/ijms24032242 (PMC9917367; doi:10.3390/ijms24032242)
Supplement: Supplementary file 1 [file ijms-24-02242-s001.zip › ijms-2148951-supplementary.pdf]

Table S1: Main criteria for inclusion

Study name	Key inclusion criteria
PROFILE1014	･18 years of age or older<br>･locally advanced, recurrent, or metastatic nonsquamous ALK-p NSCLC<br>･received no previous systemic treatment for advanced disease<br>･ECOG performance status of 0–2
PROFILE1029	･aged 18 to 70 years<br>･locally advanced, recurrent, or metastatic nonsquamous ALK-p NSCLC<br>･received no previous systemic treatment for advanced disease<br>･ECOG performance status of 0–2
ACEND-4	･18 years of age or older<br>･locally advanced or metastatic nonsquamous ALK-p NSCLC<br>･untreated with any systemic anticancer therapy<br>･WHO performance status of 0–2
ALEX	･18 years of age or older<br>･Locally advanced or metastatic ALK-p NSCLC<br>･No previous systemic treatment for advanced NSCLC<br>･ECOG performance status of 0–2
J-ALEX	･20 years of age or older<br>･Stage IIIB, IV, or postoperative recurrent ALK-p NSCLC<br>･ECOG performance status of 0–2<br>･ALK-inhibitor-naïve Japanese patients<br>･Chemotherapy naïve or who had received one previous chemotherapy regimen
ALESIA	･18 years of age or older<br>･Stage IIIB, IV ALK-p NSCLC<br>･No previous systemic treatment for advanced NSCLC<br>･ECOG performance status of 0–2
ALTA-L1	･18 years of age or older<br>･Locally advanced or metastatic ALK-p NSCLC<br>･No previous ALK-targeted therapy

CROWN	・≥ 18 or ≥ 20 years of age, according to local regulations, or older<br>・locally advanced or metastatic ALK-p NSCLC<br>・ECOG performance status score of 0–2<br>・No previous systemic treatment
eXalt3	・18 years of age or older<br>・had advanced or recurrent (stage IIIB) or metastatic (stage IV) ALK-p NSCLC<br>・ECOG performance status score of 0–2<br>・Patients may have received up to 1 prior chemotherapy regimen for metastatic disease, which may also include maintenance therapy.

yr, year; ref, reference number; ALK, anaplastic lymphoma kinase; ALK-p, anaplastic lymphoma kinase rearrangement positive; NSCLC, non-small cell lung cancer; ECOG-PS, Eastern Cooperative Oncology Group Performance Status; WHO-PS, World Health Organization performance status.

Table S2: Characteristics of included studies

Study name	Treatment arm	N	Age-yr median (range)	Female No. (%)	ECOG PS No. (%)	Histologic type No. (%)	Stage of disease at entry No. (%)	CNS metastasis No. (%)	PE	Race
PROFILE1014 2014	Pem 500 mg/m² plus Cis 75 mg/m² Carbo AUC = 5–6	171	54 (19–78)	108 (63)	0–1: 163 (95)<br>2: 8 (5)	Ade 161 (94)<br>Non-ade 10 (6)	LA 3 (2)<br>Meta 168 (98)	47 (27)	PFS	White 85 (50)<br>Asian 80 (47)<br>Other 6 (4)
	Criz 250 mg twice daily	172	52 (22–76)	104 (60)	0–1: 161 (94)<br>2: 10 (6)	Ade 161 (94)<br>Non-ade 11 (6)	LA 4 (2)<br>Meta 168 (98)	45 (26)		White 91 (53)<br>Asian 77 (45)<br>Other 4 (2)
		343/total								
ROFILE1029 2018	Pem 500 mg/ m² plus Cis 75 mg/m² Carbo AUC = 5–6	103	50 (23–69)	60 (58.3)	0–1: 99 (96.1)<br>2: 4 (3.9)	Ade 101 (98.1)<br>Lar 1 (1.0)<br>Ade-squ 1 (1.0)	LA 7 (6.8)<br>Meta 96 (93.2)	32 (31.1)	PFS	Asian 103 (100)
	Criz 250 mg twice daily	104	48 (24–67)	54 (51.9)	0–1: 100 (96.2)<br>2: 4 (3.8)	Ade 100 (96.2)<br>Large 0<br>Ade-squ 4 (3.8)	LA 13 (12.5)<br>Meta 91 (87.5)	21 (20.2)		Asian 104 (100)
		207/total								
ASCEND-4 2017	Pem 500 mg/ m² plus Cis 75 mg/m² Carbo AUC = 5–6	187	54.0 (22–80)	114 (61)	0: 70 (37)*<br>1: 105 (56)*<br>2: 11 (6)*<br>MS: 1 (1)*	Ade 183 (98)	LA 5 (3)<br>Meta 182 (97)	62 (33)	PFS	Asian 82 (44)<br>Caucasian 98 (52)<br>Other 7 (4)
	Ceri 750 mg/day orally	189	55.0 (22–81)	102 (54)	0: 69 (37)*<br>1: 107 (57)*	Ade 180 (95)	LA 9 (5)<br>Meta 180 (95)	59 (31)		Asian 76 (40)<br>Caucasian 104 (55)

					2: 13 (7)*<br>MS: 0 (0)*					Other 9 (5)
		376/total								
ALEX<br>2017	Alec 600 mg<br>twice daily	152	58 (25–88)	84 (55)	0–1: 142 (93)<br>2: 10 (7)	Ade 137 (90)<br>Squ 5 (3)<br>Other 10 (7)	IIIB 4 (3)<br>IV 148 (97)	64 (42)	PFS	Asian 69 (45)<br>Non-Asian 83 (55)
	Criz 250 mg<br>twice daily	151	54 (18–91)	87 (58)	0–1: 141 (93)<br>2: 10 (7)	Ade 142 (94)<br>Squ 2 (1)<br>Other 7 (5)	IIIB 6 (4)<br>IV 145 (96)	58 (38)		Asian 69 (46)<br>Non-Asian 82 (54)
		303/total								
J-ALEX<br>2017	Alec 300 mg<br>twice daily	103	61.0 (27–85)	62 (60)	0–1: 101 (98)<br>2: 2 (2)	Ade 100 (97)<br>Squ 2 (2)<br>Other 1 (1)	IIIB 3 (3)<br>IV 76 (74)<br>POR 24 (23)	14 (14)	PFS	JP 103 (100)
	Criz 250 mg<br>twice daily	104	59.5 (25–84)	63 (61)	0–1: 102 (98)<br>2: 2 (2)	Ade 103 (99)<br>Squ 0 (0)<br>Other 1 (1)	IIIB 3 (3)<br>IV 75 (72)<br>POR 26 (25)	29 (28)		JP 104 (100)
		207/total								
ALESIA<br>2019	Alec 600 mg<br>twice daily	125	51 (43–59)**	61 (49)	0–1: 121 (97)<br>2: 4 (3)	Ade 117 (94)	IIIB 13 (10)<br>IV 112 (90)	42 (34)	PFS	Asian 125 (100)
	Criz 250 mg<br>twice daily	62	49 (41–59)**	28 (45)	0–1: 61 (98)<br>2: 1 (2)	Ade 59 (97)	IIIB 4 (7)<br>IV 58 (94)	20 (32)		Asian 62 (100)
		187/total								
ALTA-L1	Brig 180 mg	137	58 (27–86)	69 (50)	0–1: 131 (96)	Ade 126 (92)	IIIB 8 (6)	40 (29)	PFS	Non-Asian 78 (57)

2018	once daily<br>(7-day run-in<br>period of 90 mg<br>once daily)				2: 6 (4)	Squ 4 (3)<br>Other 7 (4)	IV 129 (94)			Asian 59 (43)
	Criz 250 mg<br>twice daily	138	60 (29–89)	81 (59)	0–1: 132 (96)<br>2: 6 (4)	Ade 137 (99)<br>Squ 0 (0)<br>Other 1 (1)	IIIB 12 (9)<br>IV 126 (91)	41 (30)		Non-Asian 89 (64)<br>Asian 49 (36)
		275/total								
CROWN<br>2020	Criz 250 mg<br>twice daily	147	56 (45–66)**	91 (62)	0: 57 (39)<br>1: 81 (55)<br>2: 9 (6)	Ade 140 (95)<br>Ade-squ 5 (3)<br>Lar 1 (1)<br>Squ 1 (1)	IIIA 0 (0)<br>IIIB 8 (5)<br>IV 139 (95)<br>other 0 (0)	40 (27)	PFS	White 72 (49)<br>Asian 65 (44)<br>Black 1 (1)<br>Missing 9 (6)
	Lorl 100 mg/day<br>orally	149	61 (51–69)**	84 (56)	0: 67 (45)<br>1: 79 (53)<br>2: 3 (2)	Ade 140 (94)<br>Ade-squ 6 (4)<br>Lar 0 (0)<br>Squ 3 (2)	IIIA 1 (1)<br>IIIB 12 (8)<br>IV135 (91)<br>other 1 (1)	38 (26)		White 72 (48)<br>Asian 65 (44)<br>Black 0 (0)<br>Missing 12 (8)
		275/total								
eXalt3<br>2021	Criz 250 mg<br>twice daily	147	53 (26–90)	70 (47.6)	0–1: 139/146 (95.2)<br>2: 7/146 (4.8)	NR	IIIB 10 (6.8)<br>IV 137 (93.2)	57 (38.8)	PFS	Non-Asian 63 (42.9)<br>Asian 84 (57.1)
	Ensa 225 mg<br>once daily	143	54 (25–86)	71 (49.7)	0–1: 136 (95.1)<br>2: 7 (4.9)	NR	IIIB 13 (9.1)<br>IV 130 (90.9)	47 (32.9)		Non-Asian 66 (46.2)<br>Asian 77 (53.8)

Note; *, WHO-PS; **, Interquartile range.

yr, year; ref, reference number; N, number of patients included in the treatment arm; No., number of patients; ECOG, Eastern Cooperative Oncology Group; PS, performance status; CNS, central

nervous system; PE, primary endpoint; Pem, pemetrexed; Cis, cisplatin; Carbo, carboplatin; e3w, every 3 weeks; AUC, area under the curve; Ade, adenocarcinoma; Non-Ade, non-adenocarcinoma; LA, locally advanced; Meta, metastasis; PFS, progression-free survival; Criz, crizotinib; Lar, large; Ade-squ, adeno-squamous; Ceri, ceritinib; Alec, alectinib; POR, post-operative recurrence; JP, Japanese; squ, squamous; Brig, brigatinib; Lorl, lorlatinib; Ensa, ensartinib; NR, not reported; WHO-PS, World Health Organization performance status.

Table S3: Comparison of efficacy on PFS between generations of ALK-TKIs

Treatment comparison	HR (95% CrI)	
	ALL*	CNSM
1st vs. Chemo	0.588 (0.491–0.703)	0.800 (0.579–1.104)
2nd vs. Chemo	0.324 (0.266–0.395)	0.400 (0.283–0.566)
3rd vs. Chemo	0.164 (0.107–0.252)	0.160 (0.072–0.355)
2nd vs. 1st	0.551 (0.468–0.650)	0.501 (0.374–0.672)
3rd vs. 1st	0.280 (0.190–0.410)	0.200 (0.096–0.413)
3rd vs. 2nd	0.508 (0.334–0.771)	0.399 (0.182–0.874)

*ALL; overall patients

Comparative efficacy of each pair of treatments across four therapeutic interventions, namely, 1st, 2nd, 3rd, and Chemo, in terms of PFS for ALK-p, ALK inhibitor-naïve advanced NSCLC. Data are expressed as hazard ratios (HRs) and 95% credible intervals (CrIs) These results were also presented visually in Figure S5 in the main manuscript; PFS, progression-free survival; ALK-TKI, anaplastic lymphoma kinase tyrosine-kinase inhibitor; CNSM, central nervous system metastasis; 1st, first-generation anaplastic lymphoma kinase inhibitor (crizotinib); 2nd, second-generation anaplastic lymphoma kinase inhibitor (ceritinib, alectinib, brigatinib, and ensartinib), 3rd, third-generation anaplastic lymphoma kinase inhibitor (lorlatinib); Chemo, chemotherapy; ALK, anaplastic lymphoma kinase; ALK-p, anaplastic lymphoma kinase rearrangement positive; NSCLC, non-small cell lung cancer.

Table S4: Ranking evaluation of the efficacy of each generation of ALK-TKIs with respect to PFS

Intervention	SUCRA (rank)	
	ALL*	CNSM
Chemo	0.0 (4)	2.9 (4)
1st	33.3 (3)	30.4 (3)
2nd	66.7 (2)	67.0 (2)
3rd	100.0 (1)	99.6 (1)

*ALL; overall patients

Data presented are the surface under the cumulative ranking curve (SUCRA) for efficacy in terms of PFS for the seven therapeutic regimens (Ensa, Lorl, Brig, Alec, Ceri, Criz, and Chem) in patients with ALK-p, ALK inhibitor-naïve advanced NSCLC. Data are listed as SUCRA (%) values (rank), and higher SUCRA values indicate better outcomes; CNSM, central nervous system metastasis; PFS, progression-free survival; ALK-TKI, anaplastic lymphoma kinase tyrosine-kinase inhibitor; Ensa, ensartinib; Lorl, lorlatinib; Brig, brigatinib; Alec, alectinib; Ceri, ceritinib; Criz, crizotinib; 1st, first-generation anaplastic lymphoma kinase inhibitor (crizotinib); 2nd, second-generation anaplastic lymphoma kinase inhibitor (ceritinib, alectinib, brigatinib, and ensartinib), 3rd, third-generation anaplastic lymphoma kinase inhibitor (lorlatinib); Chemo, chemotherapy; ALK, anaplastic lymphoma kinase; ALK-p, anaplastic lymphoma kinase rearrangement positive; NSCLC, non-small cell lung cancer.

Table S5: Comparison of efficacy on PFS between ALK-TKIs and chemotherapy

Treatment comparison	HR (95% CrI)	
	ALL*	CNSM
Criz vs. Chemo	0.431 (0.348–0.533)	0.541 (0.366–0.802)
Ceri vs. Chemo	0.550 (0.417–0.725)	0.700 (0.439–1.117)
Alec vs. Chemo	0.163 (0.116–0.227)	0.200 (0.109–0.365)
Brig vs. Chemo	0.211 (0.134–0.333)	0.108 (0.044–0.267)
Lorl vs. Chemo	0.121 (0.078–0.188)	0.108 (0.048–0.249)
Ensa vs. Chemo	0.220 (0.144–0.333)	0.298 (0.145–0.612)
Ceri vs. Criz	1.278 (0.903–1.808)	1.294 (0.705–2.378)
Alec vs. Criz	0.377 (0.292–0.489)	0.369 (0.234–0.583)
Brig vs. Criz	0.490 (0.328–0.735)	0.200 (0.089–0.454)
Lorl vs. Criz	0.280 (0.191–0.412)	0.200 (0.097–0.416)
Ensa vs. Criz	0.510 (0.355–0.730)	0.550 (0.299–1.007)
Alec vs. Ceri	0.295 (0.192–0.456)	0.285 (0.133–0.612)
Brig vs. Ceri	0.384 (0.224–0.654)	0.154 (0.056–0.428)
Lorl vs. Ceri	0.219 (0.130–0.368)	0.155 (0.060–0.400)
Ensa vs. Ceri	0.399 (0.242–0.658)	0.425 (0.180–1.001)
Brig vs. Alec	1.297 (0.805–2.097)	0.542 (0.213–1.380)
Lorl vs. Alec	0.742 (0.467–1.180)	0.542 (0.230–1.285)
Ensa vs. Alec	1.351 (0.865–2.104)	1.490 (0.694–3.186)
Lorl vs. Brig	0.571 (0.327–0.999)	0.999 (0.334–2.991)
Ensa vs. Brig	1.041 (0.604–1.788)	2.753 (0.992–7.595)
Ensa vs. Lorl	1.821 (1.072–3.071)	2.747 (1.061–7.051)

*ALL; overall patients

Comparison of efficacy—in terms of PFS—of each pair of treatments across seven therapeutic regimens, including Ensa, Lorl, Brig, Alec, Ceri, Criz, and Chemo for ALK-p, ALK-inhibitor naïve-advanced NSCLC for overall participants and in subgroup with CNSM. Data are expressed as hazard ratios (HRs) and 95% credible intervals (CrIs); CNSM, central nervous system metastasis; PFS, progression-free survival; ALK-TKI, anaplastic lymphoma kinase tyrosine-kinase inhibitor; Ensa, ensartinib; Lorl, lorlatinib; Brig, brigatinib; Alec, alectinib; Ceri, ceritinib; Criz, crizotinib; Chemo, chemotherapy; ALK, anaplastic lymphoma kinase; ALK-p, anaplastic lymphoma kinase rearrangement positive; NSCLC, non-small cell lung cancer.

Table S6: Ranking evaluation of the efficacy of ALK-TKIs with respect to PFS

Interventions	SUCRA (rank)	
	ALL*	CNSM
Chemo	0.0 (7)	1.2 (7)
Criz	32.0 (5)	30.4 (5)
Ceri	18.1 (6)	19.4 (6)
Alec	81.2 (2)	67.2 (3)
Brig	62.1 (3)	89.5 (2)
Lorl	97.6 (1)	90.0 (1)
Ensa	59.1 (4)	52.4 (4)

*ALL; overall patients

Data presented are the surface under the cumulative ranking curve (SUCRA) for efficacy regarding PFS of the seven therapeutic regimens (Ensa, Lorl, Brig, Alec, Ceri, Criz, and Chemo) in patients with ALK-p, ALK-inhibitor naïve-advanced NSCLC in overall participants and in subgroup with CNSM. The data are listed as SUCRA values (rank) and higher SUCRA values indicate better outcomes. PFS, progression-free survival; ALK-TKI, anaplastic lymphoma kinase tyrosine-kinase inhibitor; Ensa, ensartinib; Lorl, lorlatinib; Brig, brigatinib; Alec, alectinib; Ceri, ceritinib; Criz, crizotinib; Chemo, chemotherapy; ALK, anaplastic lymphoma kinase; ALK-p, anaplastic lymphoma kinase rearrangement positive; NSCLC, non-small cell lung cancer; CNSM, central nervous system metastasis.

Table S7: Comparison of efficacy on PFS between ALK-TKIs and chemotherapy by race

Treatment comparisons	HR (95% CrI)	
	non-asian	asian
Criz vs. Chemo	0.529 (0.364–0.772)	0.418 (0.323–0.540)
Ceri vs. Chemo	0.440 (0.297–0.653)	0.660 (0.411–1.062)
Alec vs. Chemo	0.259 (0.147–0.457)	0.138 (0.091–0.209)
Brig vs. Chemo	0.286 (0.153–0.534)	0.171 (0.079–0.371)
Lorl vs. Chemo	0.101 (0.053–0.194)	0.196 (0.107–0.363)
Ensa vs. Chemo	0.323 (0.160–0.649)	0.134 (0.074–0.241)
Ceri vs. Criz	0.832 (0.484–1.433)	1.581 (0.923–2.713)
Alec vs. Criz	0.490 (0.320–0.750)	0.330 (0.239–0.457)
Brig vs. Criz	0.540 (0.328–0.893)	0.410 (0.198–0.852)
Lorl vs. Criz	0.190 (0.112–0.325)	0.470 (0.270–0.821)
Ensa vs. Criz	0.610 (0.337–1.100)	0.320 (0.188–0.543)
Alec vs. Ceri	0.589 (0.295–1.175)	0.209 (0.112–0.392)
Brig vs. Ceri	0.649 (0.309–1.361)	0.259 (0.104–0.644)
Lorl vs. Ceri	0.229 (0.107–0.490)	0.298 (0.137–0.646)
Ensa vs. Ceri	0.733 (0.329–1.632)	0.202 (0.095–0.431)
Brig vs. Alec	1.103 (0.571–2.129)	1.240 (0.560–2.759)
Lorl vs. Alec	0.388 (0.196–0.769)	1.422 (0.750–2.710)
Ensa vs. Alec	1.246 (0.599–2.578)	0.969 (0.519–1.801)
Lorl vs. Brig	0.351 (0.169–0.734)	1.146 (0.456–2.868)
Ensa vs. Brig	1.129 (0.519–2.453)	0.781 (0.316–1.923)
Ensa vs. Lorl	3.207 (1.442–7.083)	0.680 (0.315–1.458)

Comparison of efficacy—in terms of PFS—of each pair of treatments across seven therapeutic regimens, including Ensa, Lorl, Brig, Alec, Ceri, Criz, and Chemo for ALK-p, ALK-inhibitor naïve-advanced NSCLC for non-asian and asian subgroup. Data are expressed as hazard ratios (HRs) and 95% credible intervals (CrIs); PFS, progression-free survival; ALK-TKI, anaplastic lymphoma kinase tyrosine-kinase inhibitor; Ensa, ensartinib; Lorl, lorlatinib; Brig, brigatinib; Alec, alectinib; Ceri, ceritinib; Criz, crizotinib; Chemo, chemotherapy; ALK, anaplastic lymphoma kinase; ALK-p, anaplastic lymphoma kinase rearrangement positive; NSCLC, non-small cell lung cancer.

Table S8: Ranking evaluation of the efficacy of ALK-TKIs with respect to PFS

Interventions	SUCRA (rank)	
	non-asian	asian
Chemo	0.0 (7)	0.7 (7)
Criz	21.9 (6)	32.7 (5)
Ceri	36.1 (5)	16.8 (6)
Alec	71.2 (2)	83.6 (2)
Brig	64.6 (3)	70.0 (3)
Lorl	99.9 (1)	61.4 (4)
Ensa	56.4 (4)	84.8 (1)

Data presented are the surface under the cumulative ranking curve (SUCRA) for efficacy regarding PFS of the seven therapeutic regimens (Ensa, Lorl, Brig, Alec, Ceri, Criz, and Chemo) in patients with ALK-p, ALK-inhibitor naïve-advanced NSCLC in non-asian and asian subgroup. The data are listed as SUCRA values (rank) and higher SUCRA values indicate better outcomes. PFS, progression-free survival; ALK-TKI, anaplastic lymphoma kinase tyrosine-kinase inhibitor; Ensa, ensartinib; Lorl, lorlatinib; Brig, brigatinib; Alec, alectinib; Ceri, ceritinib; Criz, crizotinib; Chemo, chemotherapy; ALK, anaplastic lymphoma kinase; ALK-p, anaplastic lymphoma kinase rearrangement positive; NSCLC, non-small cell lung cancer.

Table S9: Sensitivity analysis for patients with no previous systemic anti-cancer therapy

Treatment comparison	HR (95% CrI)
1st vs. Chemo	0.592 (0.494–0.708)
2nd vs. Chemo	0.320 (0.262–0.391)
3rd vs. Chemo	0.165 (0.108–0.253)
2nd vs. 1st	0.541 (0.455–0.644)
3rd vs. 1st	0.280 (0.190–0.410)
3rd vs. 2nd	0.518 (0.339–0.789)

A sensitivity analysis was performed by excluding the group of patients with prior treatment with systemic anticancer chemotherapy included in three trials (J-ALEX, ALTA-1L, and eXalt3) and including only patients with no prior treatment with systemic anticancer therapy. Comparative efficacy for PFS of each pair of treatments across the four therapeutic regimens, including first-generation ALK inhibitor (Criz), second-generation ALK inhibitor (Ensa, Brig, Alec, and Ceri), third-generation ALK inhibitor (Lorl), and Chemo for ALK-p, ALK inhibitor-naïve advanced NSCLC, is shown. Data are expressed as hazard ratios (HRs) and 95% credible intervals (CrIs); PFS, progression-free survival; Ensa, ensartinib; Lorl, lorlatinib; Brig, brigatinib; Alec, alectinib; Ceri, ceritinib; Criz, crizotinib; 1st, first-generation anaplastic lymphoma kinase inhibitor (crizotinib); 2nd, second-generation anaplastic lymphoma kinase inhibitor (ceritinib, alectinib, brigatinib, and ensartinib), 3rd, third-generation anaplastic lymphoma kinase inhibitor (lorlatinib); Chemo, chemotherapy; ALK, anaplastic lymphoma kinase; ALK-p, anaplastic lymphoma kinase rearrangement positive; NSCLC, non-small cell lung cancer.

Table S10: Sensitivity analysis of PFS assessment rankings in patients with no previous systemic anticancer therapy

Intervention	SUCRA (rank)
Chemo	0.0 (4)
1st	33.3 (3)
2nd	66.7 (2)
3rd	100.0 (1)

A sensitivity analysis was performed by excluding the group of patients with prior treatment with systemic anticancer chemotherapy included in three trials (J-ALEX, ALTA-1L, and eXalt3) and including only patients without any prior treatment with systemic anticancer therapy. Data presented are the surface under the cumulative ranking curve (SUCRA) for efficacy for PFS of each treatment across the four therapeutic regimens, including first-generation ALK inhibitor (Criz), second-generation ALK inhibitor (Ensa, Brig, Alec, and Ceri), third-generation ALK inhibitor (Lorl), and Chemo in patients with ALK-p, ALK inhibitor-naïve advanced NSCLC. The data are listed as SUCRA(%) values (rank) and higher SUCRA values indicate better outcomes.

PFS, progression-free survival; Ensa, ensartinib; Lorl, lorlatinib; Brig, brigatinib; Alec, alectinib; Ceri, ceritinib; Criz, crizotinib; 1st, first-generation anaplastic lymphoma kinase inhibitor (crizotinib); 2nd, second-generation anaplastic lymphoma kinase inhibitor (ceritinib, alectinib, brigatinib, and ensartinib), 3rd, third-generation anaplastic lymphoma kinase inhibitor (lorlatinib); Chemo, chemotherapy; ALK, anaplastic lymphoma kinase; ALK-p, anaplastic lymphoma kinase rearrangement positive; NSCLC, non-small cell lung cancer.

Figure S1:

Risk of bias domains

Study	D1	D2	D3	D4	D5	overall
PROFILE1014	+	-	+	-	+	-
PROFILE1029	-	-	+	-	+	-
ASCEND-4	+	-	+	-	+	-
ALEX	+	-	+	-	+	-
J-ALEX	+	-	+	-	+	-
ALESIA	+	-	+	-	+	-
ALTA-1L	+	-	+	-	+	-
CROWN	+	-	+	-	+	-
eXalt3	+	-	+	-	+	-

Domains:
D1: Bias arising from the randomization process
D2: Bias due to deviations from intended interventions
D3: Bias due to missing outcome data
D4: Bias in measurement of the outcome
D5: Bias in selection of the reported result

Figure S1: Risk of bias summary. The author's assessment of each risk of bias item for each incorporated study is expressed. The symbols "+," "-," and "×" indicate a low risk of bias, some concerns, and a high risk of bias, respectively. The quality of the included studies was considered generally good, as no study was assessed as having a high risk of bias. However, the nine studies in this systematic review and the NMA were all judged to be some concerns in the overall analysis. This is most likely because these studies were open-label trials judged as some concerns in the area of bias due to deviations from the intended intervention or bias in the measurement of outcomes. In addition, PROFILE 1029 was judged to be some concerns in the area of bias arising from randomization because this process was not sufficiently detailed; NMA, network meta-analysis.

Figure S2:

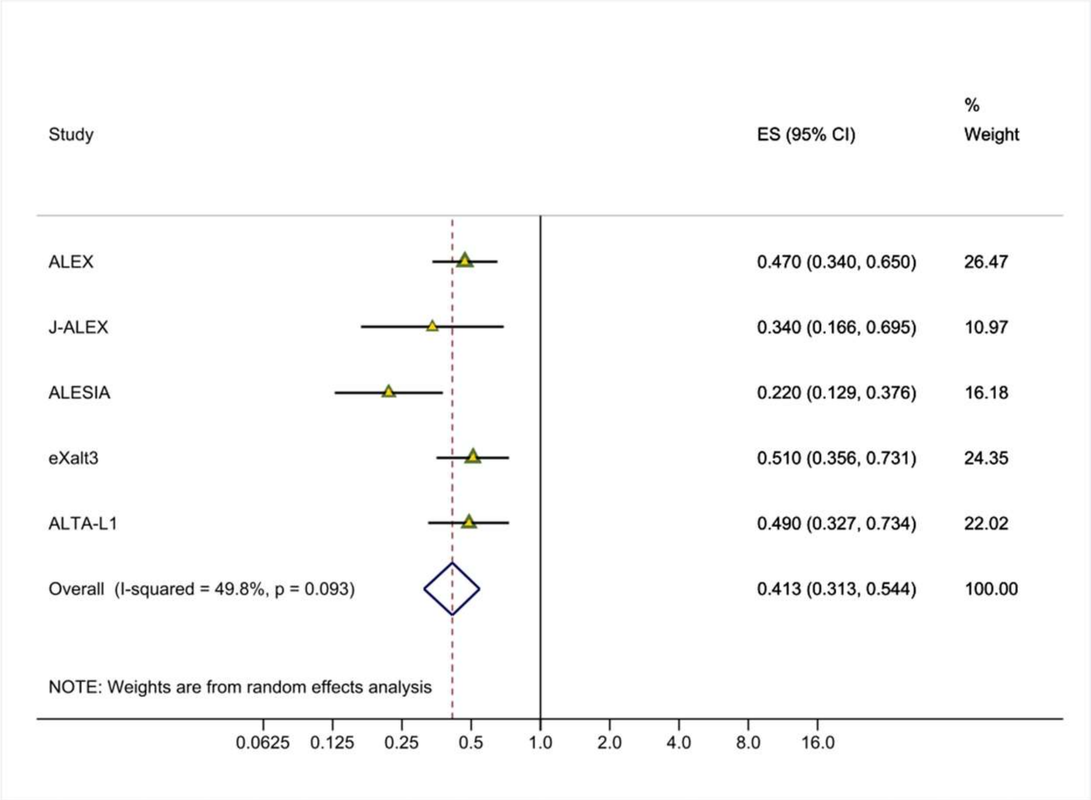

Figure S2: Forest plot of five trials comparing second-generation ALK inhibitors (alectinib, brigatinib, and ensartinib) and crizotinib. A meta-analysis of five trials (ALEX, J-ALEX, ALESIA, eXalt3, and ALTA-L1) comparing second-generation ALK inhibitors and Criz for PFS was performed based on a random-effect model, with an assessment of heterogeneity being the main objective. Heterogeneity (I^2) was expressed as I-squared (%). Overall effect size (ES) for PFS was expressed as HR and 95% CI. Data have been obtained from previous studies; PFS, progression-free survival; ALK, anaplastic lymphoma kinase; HR, hazard ratio; CI, confidence interval; Criz, crizotinib.
